# Evolution of Public Health Expenditure Financed by the Romanian Social Health Insurance Scheme From 1999 to 2019

**DOI:** 10.3389/fpubh.2021.795869

**Published:** 2021-12-01

**Authors:** Ciprian-Paul Radu, Bogdan Cristian Pana, Daniel Traian Pele, Radu Virgil Costea

**Affiliations:** ^1^Department of Public Health, University of Medicine and Pharmacy “Carol Davila”, Bucharest, Romania; ^2^Roche Romania Ltd., Bucharest, Romania; ^3^Faculty of Cybernetics, Statistics and Economic Informatics, University of Economic Studies, Bucharest, Romania; ^4^Department of General Surgery 2, University of Medicine and Pharmacy “Carol Davila”, Bucharest, Romania

**Keywords:** financing, trends, health system, health budget, hospital budget, drug expenditure

## Abstract

The Romanian health system is mainly public financed (80.45%) through the following sources: Social Health Insurance (65%), State and Local Authorities Budget (15.45%), while the private sources (voluntary health insurance and out of pocket) adds an additional 19.55% to the public funds. The shares of the types of expenditure reflect the importance of each sector in the overall health system, and trends in expenditure show the impact of financing on the health sector's structural changes. We analyzed the 20-year trend of the Social Health Insurance budget, from 1999 to 2019. The influences of the different allocations, subcategories, and new budget categories appearing over time were adjusted to reveal relevant trends. Of the 14 medical service categories and the stand-alone *Administrative expenditure* category, six expenditure categories including *Hospital services, Total drugs*, and *Primary care* showed stationary 20-year trends; five including *Medical devices, Dialysis*, and *Homecare services* showed ascendant trends; and four including *Dentistry* and *Emergency services* showed descendant trends. Stationary trends imply no structural changes in the health sector of relevant magnitude to impact the financing shares of major categories: hospitals, drugs, or primary care. Emerging trends related to the impact of different reforms were revealed only in the low share of expenditures categories. The allocation methodology and statistical analysis of the trends reveal a new perspective on the evolution of health sector in Romania.

## Introduction

The Social Health Insurance (SHI) scheme was introduced in Romania in 1999 and represents the health care system's main source of funding and is managed by the National Health Insurance House. It accounted for ~80.8% of public spending on health care in 2019, while direct public funding from the State Budget, Local Authorities and Ministry of Health covered the remaining 19.2%. Public spending on health care exceeded 10 billion Euros and private spending over 2 billion Euros in 2019 (1), marking a significant increase over the last 20 years (e.g., total public health expenditure was 1.6 billion US$ in 2001) (2). However, Romania remains one of the countries with the lowest percentage of Gross Domestic Product allocated to health care, 5.16%, and with one of the lowest values of health expenditure per capita, 494 Euro in 2017^1^.

Over the last 20 years several reforms were carried out in various domains of the health care system. Reforms with an impact on public health include structural changes in health services with the objective of increasing community services at the grassroot and family medicine level and decreasing tertiary care services; the National Health Programs launched by the Ministry of Health; improvements in primary health and emergency care; payment of the hospitals on a case-by-case basis (DRG system); the improvement of hospital management; the introduction of co-payments; the introduction of health technology assessment for drugs; the increase of wages for medical staff in public hospitals etc. (2, 3).

We analyzed the yearly evolution of public health expenditure financed by the SHI over a 20-year period and compared the trends with the realities in the health care system. To make the comparison relevant, we allocated each expenditure category to the right type of health care service and used statistical techniques to reveal trends and break points. The SHI expenditure is spliced (by type of health care services) yearly in the Romanian Parliament through the vote for the State Budget Law; hence, we consider that this budget allocation represents the main influencer of the activities of each provider of health care services (provision of services being driven by the budget available rather than the demand for health care services). We believe that our study makes a significant contribution to the literature with the longitudinal approach of the trends of SHI expenditures per type of health care services. This brings another perspective of the health reforms from a financier lens, applying the axiom that services follow the money in Romania.

## Methods

We reviewed public documents and literature from 1999 to 2019 on the expenditures of SHI, the State Budget and Ministry of Health, Territorial Administrative Units (TAU), health system reform, and related legislation. Various analyses were performed:

- Analysis of legislation related to social services and health care from 1997 to 2019 (Social Insurance Health Law being voted in 1997);
- Analysis of various public documents of the Ministry of Health (MoH), Ministry of Interior, Ministry of Finance, National Commission for Strategy and Prognosis, National Institute of Statistics, and National Romanian Bank, referring to public health expenditure from 1999 to 2019;
- Analysis of SHI expenditure and analysis of trends for categories of SHI expenditure.

SHI collects its income (revenues) mainly from monthly contributions of employers and employees', and contributions from free-lancers and subsidies from the State Budget. The income is used to cover the Health and social care expenses. A Reserve fund could be created only if the health insurance income is higher than the health and social care expenditure. Each year the SHI scheme has a surplus/deficit, based on the income and expenditure levels (including reserve fund).

The SHI expenditure was broken down as follows:

◦ **Health expenditure** (which include all type of medical services expenditures)
◦ **Administrative expenditure** [expenditures of the National Health Insurance House (NHIH) and the Local Health Insurance Houses (LHIH) for their own functioning]
◦ **Social care expenditures** (since 2006, to cover expenditures for sick leave, impairment of work capacity etc.)

Medical services expenditures were analyzed based on the types of expenditure categories related to various health service providers as they appeared with funds allocated in the annual state budget from 1999 to 2019 (4).

The evolution of the social care expenditures that appeared in the SHI expenditure structure in 2006 was not analyzed as it reflects the actions at the social care system level rather than the health care system level.

When SHI began in 1999, there were only nine types of medical expenses for the main categories of health service providers. By 2019 another seven types of medical expenses were added, arriving at the following 16 categories:

1. *Primary care* (family physicians)
2. *Multifunctional medical centers* (small outpatient facilities)
3. *Dentistry* (for dental care)
4. *Emergency services* (pre-hospital medical services like ambulance and emergency care units from some hospitals)
5. *Medical devices* (different medical devices like prosthesis, hearing devices etc.)
6. *Outpatient drugs* (drugs dispensed from community pharmacies)
7. *Drugs within the Curative National Health Programs* (NHPs) (Prior to 2006, they were reported together with *Sanitary Materials from the National Curative Health Programs*. Considering the share of 9:1 calculated after 2006 between *Drugs within the Curative NHPs* and *Sanitary Materials from the Curative NHPs*, 90% of the total expenditure on medicine and sanitary materials from the NHPs were allocated to the expenditure on *Drugs within the Curative NHPs*, and 10% were allocated to expenditure on *Sanitary Materials from the Curative NHPs*)
8. *Sanitary materials from the Curative NHPs*, covering different materials like tests, kits for diagnosis, heart replacement valves, prosthesis etc. (reported separately since 2006—see above)
9. *Outpatient services* (clinical outpatient medical services, inclusive of laboratory services prior to 2001)
10. *Paraclinical (laboratory) services* (since 2001)
11. *Hospital services* (in-patient care)
12. *Dialysis* (since 2002)
13. *Rehabilitation hospitals* (rehabilitation in-patient services since 2006)
14. *Home Care services* (since 2003)
15. *Medical services received in the European Union (EU)* (since 2007)
16. *Transfers to public units* (since 2016)—represents funds transferred directly from the SHI budget to hospitals to cover salary increases for physicians and nurses practicing in public hospitals.

To reflect the public expenditure on these 16 categories of expenditure as accurately as possible, the following adjustments of the primary data were made:

### Adjustment on Drugs Expenditure

#### Total Drugs Expenditure

There are several sources of expenditure for drugs, two of them being *Outpatient drugs* and *Drugs within the Curative NHPs*. However, the expenditure for drugs in hospital services is not considered in the above-mentioned categories.

Within Diagnostic Related Group (DRG) payment mechanisms for hospital services, all types of expenses are covered, including medicines. Approximately 8–10% of the total drug consumption recorded for claw-back, from 2012, was the consumption of drugs in hospitals (5). Therefore, we extracted this expenditure for drugs from *Hospital services* and added it to *Total drugs* expenditure.

Having made this adjustment, we created a category of expenditure named *Total drugs* expenditure which included all the categories (*Outpatient drugs* and *Drugs within the Curative NHPs)* and the drugs expenses deducted from the *Hospital services* category. The rationale for this adjustment lies in the willingness to provide a clear picture on the trend of expenditures for defined categories, which have different incentives and regulations within the health care system (e.g., drugs used in hospitals have the same regulations and providers like the drugs in outpatient pharmacies).

#### Claw-Back

According to the Government Emergency Ordinance (GEO) # 77/2011, the marketing authorization holder (MAH) of each drug must pay a quarterly contribution related to drug consumption (claw-back taxation introduced from Q4-2011). This tax is calculated as the difference between quarterly consumption of medicines registered by the NHIH and the quarterly budget set by the GEO # 77/2011. The claw-back values ranged from a minimum of 12.35% (in Q3-2015) to a maximum of 27.65% (in Q4-2019). These amounts paid by the MAH are regarded in our analysis as a discount on drugs expenditure, because they reflect a reduction in the price of medicines; thus, lowering the costs for the *Total drugs* expenditure category. Consequently, the claw-back payments were deducted from *Total drugs* expenditure and from *Total SHI expenditure*, because they were not public funding sources, but private contributions for health care financing.

#### Historical Debts

During the period under review, a large variation in drug expenditure was observed between 2011 and 2014, with a peak in 2013, without certain reform measures being taken in that year to justify the high increase in drug expenditure. The apparent explanation was that a payment of arrears (debts older than 90 days) of over 2 billion RON for drugs was made in 2013. Therefore, the high expenditure in 2013 came from 2012 consumption (6). For the analysis of the annual evolution of the different types of expenditure, we moved the 2 billion RON excess of 2013–2012.

### Adjustment on Hospital Services Expenditure

As mentioned earlier, *Hospital services* expenditure was reduced by the deduction of the drugs expenses, which were transferred to the *Total drugs* expenditur*e* category, for the entire 1999–2019 period.

#### Transfers to Public Units

This expenditure, started in 2016, was a supplementary payment to public hospitals dedicated to covering medical personnel's salary increases. It was added to *Hospital services* because these expenses were intended to cover part of the expenses for medical personnel salaries from the hospitals.

### Adjustment for Currency Exchange

#### Currency Conversion From RON to EURO

When analyzing the evolution of the total amounts spent by the NHIH in the 1999–2019 period, amounts expressed in Euro were used based on the conversion of amounts from the local Romanian currency RON to Euro at the average exchange rate of the applicable year (7).

### Testing the Statistical Significance of the Trends in SHI Expenditure Categories

We used the Kwiatkowski–Phillips–Schmidt–Shin (KPSS) test to identify the existence of a deterministic trend for each time series item (8). The hypotheses of the test are the following:


$$\begin{array}{r} \{\begin{array}{c} H_{0}:Y_{t}\;\sim \;I\left(0\right)\to trend\;stationary\\ H_{1}:Y_{t}\;\sim \;I\left(1\right)\to difference\;stationary \end{array} \end{array}$$

Basically, for each time series *Y*_*t*_, we estimated the regression model *Y*_*t*_ = α + β*t* + *a*_*t*_ and we estimated the regression residuals *e*_*t*_ = *Y*_*t*_ − Ŷ_*t*_, where ${Ŷ}_{t}=\hat{\alpha }+\hat{\beta }t$.

The test statistic is $KPSS=n^{-2}\overset{n}{\underset{t=1}{\sum }}\frac{S_{t}}{{\hat{\sigma }}^{2}}$, where ${\hat{\sigma }}^{2}$ is the estimated variance of residuals and $S_{t}=\overset{t}{\underset{i=1}{\sum }}e_{i}$.

The results of the KPSS test are reported in **Table 2**.

We used the Chow breakpoint test to test for the existence of some structural breaks in each time series, based on the regression model described above (9). All KPSS test and Chow breakpoint test results are listed in **Tables 2, 3** in section Results.

## Results

### Evolution of SHI Expenditures in Absolute Amounts

The initial years of SHI operation (1999–2003) had the largest surplus of funds. However, after 2003, due to deficits in the NHIH budget, Law No. 95/2006 was adjusted to permit the State Budget to supplement funds.

In absolute amounts, the SHI expenditure grew almost eight times during 1999–2019.

**Figure 1:**
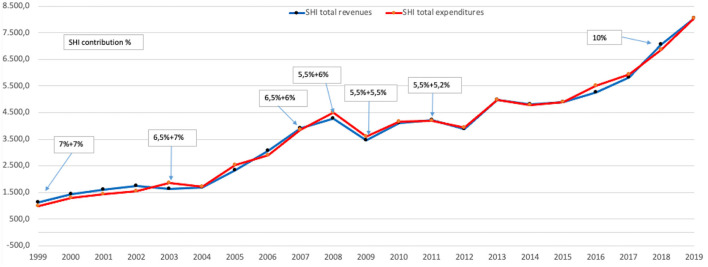
Evolution of SHI expenditures and revenues 1999–2019, million Euro.

The only years of decline or stagnation in spending were the years 2009–2012 due to the global economic crisis (Figure 1). After 2015, the expenditure almost doubled in just 5 years. All categories of expenditure had increased about 5–15 times, except for *Dentistry* and *Multifunctional medical centers* which had only increased about 1–2 times.

In the period 1999–2019 the contribution from salaries to SHI declined continuously, e.g., from 14% (7% calculated from the gross salary, paid by the employer + 7% calculated from the gross salary paid by the employee, in 1999) to 10.7% (5.5% employer + 5.2% employee, in 2011) and 10% (10% from employee, in 2018) (10). The increase of the absolute amounts for public financing, despite the decrease in the contribution rate, shows the growth of the economic situation in Romania in the last two decades.

Of the ~8 billion Euro spent by SHI on health services in the year 2019, more than 2 billion Euro was in the category of *Transfers to public units*. The amount of 2 billion Euro is roughly equal to that spent by hospitals to cover all their expenditure, and by merging the *Transfers to public units* and hospital expenditures (as explained previously), the *Hospital services* category represented the main consumer of resources in 2019, using over 55% of the SHI budget.

The *Hospital services* category has been the main expenditure category since 1999 (55.8% of the SHI budget), except for the years 2011 to 2013 when it was at its lowest level of 36.4% of the SHI budget, and was surpassed by the *Total drugs* expenditure category. This decrease of hospital expenditures could be the impact of the closure of 67 hospitals in 2011 (subsequently re-opened one by one), which could be a good example of reform course modified by the political decision (11).

### Evolution of the Shares of Different Types of Expenditure in SHI Budget

The trend of evolution of different types of expenditure between 1999 and 2019 was analyzed for the 16 categories of medical services validated using the statistical tests presented above. As we presented in the Methods section, the *Outpatient drugs* and *Drugs within the Curative NHPs* categories were merged in the new *Total drugs* category and the *Transfers to public units* category was included in the *Hospital services* category resulting in a total of 14 medical service categories, together with Administrative expenditure as a stand-alone category (Table 1).

**Table 1:** The share of different categories of health care provision in SHI expenditure between 1999 and 2019.

**Year**	**Primary care (%)**	**Multifunctional. centers (%)**	**Outpatient services (%)**	**Para clinic (%)**	**Dentistry (%)**	**Materials in NHPs (%)**	**Medical devices (%)**	**Emergency services (%)**	**Rehabilitation (%)**	**Dialysis (%)**	**Homecare (%)**	**EU medical services (%)**	**Admin. expend. (%)**	**Hospital services (%)**	**Total drugs (%)**
1999	8.8	1.1	6.0	0.0	2.4	0.5	0.3	3.5	0.0	0.0	0.0	0.0	3.0	55.8	18.6
2000	8.6	0.5	7.3	0.0	1.3	0.8	0.3	2.9	0.0	0.0	0.0	0.0	2.8	49.9	25.6
2001	7.1	0.6	3.9	2.7	1.3	0.9	0.5	2.9	0.0	0.0	0.0	0.0	2.1	50.3	27.7
2002	6.5	0.8	3.6	2.5	1.1	0.8	0.5	2.8	0.0	1.6	0.0	0.0	1.8	51.7	26.4
2003	5.0	1.1	3.0	2.2	0.8	0.8	0.4	2.8	0.0	2.3	0.0	0.0	2.6	51.5	27.2
2004	5.0	1.1	2.8	2.9	0.8	0.8	0.5	3.1	0.0	2.4	0.0	0.0	1.5	49.8	29.3
2005	5.0	1.0	2.7	2.4	0.7	0.7	0.5	2.9	0.0	1.9	0.0	0.0	1.3	46.2	34.6
2006	4.8	0.5	2.7	2.3	0.6	0.7	0.5	3.0	0.5	3.9	0.0	0.0	1.7	45.6	33.1
2007	6.1	0.6	2.7	3.5	0.5	0.7	0.7	3.0	0.5	3.2	0.1	0.0	2.2	43.4	32.8
2008	9.2	0.6	2.5	3.8	0.5	0.7	0.7	3.5	0.4	2.6	0.1	0.1	1.6	42.3	31.5
2009	7.7	0.5	2.2	2.3	0.4	0.9	0.7	4.3	0.5	3.4	0.1	0.1	1.5	45.5	29.9
2010	6.7	0.4	1.8	1.9	0.4	0.6	0.5	4.0	0.4	3.5	0.2	0.1	2.3	46.1	31.3
2011	6.6	0.4	1.8	1.9	0.3	0.8	0.6	3.9	0.3	3.9	0.2	0.1	1.8	37.6	39.9
2012	6.5	0.4	2.1	1.7	0.3	0.6	0.6	3.5	0.3	3.7	0.2	0.3	1.9	36.4	41.6
2013	7.0	0.4	2.9	1.9	0.1	1.1	0.8	0.6	0.3	3.8	0.2	0.8	1.6	38.5	40.1
2014	7.1	0.4	3.1	2.8	0.2	1.1	0.9	0.2	0.2	3.9	0.3	1.6	1.6	38.5	38.2
2015	7.4	0.5	3.2	3.1	0.4	1.2	0.9	0.2	0.3	4.2	0.3	2.0	1.2	40.0	35.2
2016	6.7	0.4	3.4	2.8	0.3	1.6	0.9	0.2	0.3	4.0	0.2	2.5	1.0	43.4	32.3
2017	6.6	0.5	3.6	2.9	0.3	1.4	0.8	0.1	0.2	3.8	0.2	2.0	1.4	52.8	23.5
2018	6.7	0.4	3.8	2.5	0.3	1.1	0.7	0.1	0.2	3.3	0.1	1.5	1.1	57.2	21.1
2019	7.3	0.3	3.7	2.2	0.3	1.3	0.6	0.1	0.1	3.1	0.1	1.3	1.2	54.7	23.6

KPSS test and Chow breakpoint test results are listed in Tables 2, 3.

**Table 2:** KPSS statistics test for the trends in different types of expenditure, 1999–2019.

**Type of expenditure**	**KPSS test statistic**	**Type of time series**	**Confidence level for stationarity**
Primary care	0.086	Stationary	^***^
Multifunctional medical centers	0.076	Trend stationary—descendent trend	^***^
Dentistry	0.199	Trend stationary—descendent trend	^***^
Emergency services	0.129	Trend stationary—descendent trend	^**^
Medical devices	0.085	Trend stationary—ascendant trend	^***^
Total drugs	0.160	Stationary	^***^
Sanitary materials from NHP	0.138	Trend stationary—ascendant trend	^**^
Outpatient services	0.190	Stationary	^***^
Paraclinical (laboratory) services	0.121	Stationary	^**^
Hospitals services	0.165	Stationary	^***^
Dialysis	0.190	Trend stationary—ascendant trend	^***^
Rehabilitation hospitals	0.143	Stationary	^**^
Home care services	0.124	Trend stationary—ascendant trend	^**^
Medical services received in EU	0.130	Trend stationary—ascendant trend	^**^
Administrative expenditure	0.097	Trend stationary—descendent trend	^***^

*** *99%*,

** *95%—using EViews 10*.

**Table 3:** Chow breakpoint test results for the trends in different types of expenditure, 1999–2019.

**Type of expenditure**	**Chow breakpoint test—*F* statistic**	**Year of structural break**	***P*-value**	**Confidence level**
Primary care	12.952	2008	0.000	^***^
Multifunctional medical centers	NA	NA	NA	NA
Dentistry	7.690	2013	0.004	^***^
Emergency services	96.977	2013	0.000	^***^
Medical devices	NA	NA	NA	NA
Total drugs	35.937	2012	0.000	^***^
Sanitary materials from NHP	5.728	2016	0.013	^**^
Outpatient services	18.752	2010	0.000	^***^
Paraclinical (laboratory) services	5.149	2008	0.018	^**^
Hospitals services	42.113	2012	0.000	^***^
Dialysis	9.243	2015	0.002	^***^
Rehabilitation hospitals	NA	NA	NA	NA
Home care services	18.612	2015	0.000	^***^
Medical services received in EU	6.015	2016	0.011	^**^
Administrative expenditure	0.171	2016	0.844	Not significant

*** *99%*,

** *95%—using EViews 10*.

For KPSS test, the null hypothesis is that the time series is trend stationary (TS) vs. the unit root alternative time series is stationary. The test was performed in EViews 10.

The Chow breakpoint test was conducted using EViews 10, and we considered significant the results with *p*-values lower than 0.05.

### Categories of Services With Stationary Trends

#### Hospital Services Expenditure

- As described in section Methods, *Hospital services* expenditure category includes also the *Transfers to public units* category expenditure (that reached a share of over 24% in 2018 and 2019). *Hospital services* expenditure had a share of 54.7% in 2019 compared with 55.8% in 1999, despite declarations and reform measures that should foster primary care and outpatient services.
- An analysis on the *Hospital services* expenditure category revealed that the 20-year evolutionary trend was significantly stationary (KPSS 0.165, Confidence level (Cl) 99%) and there was a significant breakpoint from 2012 (*p* < 0.001).

#### Total Drugs Expenditure

- The *Total drugs* expenditure, adjusted as presented in section Methods, grew the fastest from 1999 until 2012 when the claw-back mechanism was introduced and the trend then changed (breakpoint in 2012, *p* < 0.001). Despite this breakpoint, the *Total drugs* expenditures category evolutionary trend was stationary, with yearly adjustments given by the annual price corrections, exchange rate variation, and introduction of a separate budget for medicines conditionally included in the *Reimbursed Drug List* through cost-volume/cost-volume-result contracts.
- An analysis on the *Total drugs* expenditure category revealed that the 20-year evolutionary trend was significantly stationary (KPSS 0.160, Cl 99%).

#### Rehabilitation Hospitals

- The share of *Rehabilitation hospitals* is very small in SHI budget (below 0.5%) and reflects the low importance of this category of health care.
- An analysis on the *Rehabilitation hospitals* expenditure revealed that the 20-year evolutionary trend was significantly stationary (KPSS 0.143, Cl 95%).

#### Primary Care Expenditure

- Although it is at a lower value (below 9.2%), the expenditure for *Primary care* (family physicians) remained stationary (KPSS 0.086, Cl 99%) with a significant breakpoint in 2008 (*p* < 0.001) when a special program for evaluation of the health status of the Romanian population increased expenditure. Unfortunately, the program did not generate specific areas of intervention and effective measures to tackle existing health problems.

#### Outpatient Services

- The O*utpatient services* expenditure covers the outpatient visits provided by physicians outside hospitals and its share was between 7.3% in 2000, decreasing to 1.8% in 2010, to increase again to 3/7% in 2019. 2010 is the significant breakpoint, *p* < 0.001.
- An analysis on the O*utpatient services* expenditure revealed that the 20-year evolutionary trend was significantly stationary (KPSS 0.190, Cl 99%).

#### Paraclinical (Laboratory) Services

- *Paraclinical (laboratory) services* had an average of 2.5% of the budget over the last decade. Initially, it increased to a maximum of 3.8% in 2008 (significant breakpoint, *p* < 0.05) and then decreased in the next period. Thus, the 20-year evolutionary trend for *Paraclinical (laboratory) services* expenditure was significantly stationary (KPSS 0.121, Cl 95%).

*Primary Care* expenditure together with expenditure for *Paraclinical (laboratory) services* and O*utpatient services* should show synchronization with the health sector reform policy reflected in the Strategy of 2014–2020 to increase the role of primary medicine. However, the outpatient care sector remained in the same allocation range of 12–15% over 20 years, without a synchronization of the allocation of funds with the policy of stimulating primary medicine. Thus, we did not observe a “reverse of the pyramid of services” from hospitals to outpatient care (12).

### Categories of Services With Ascendant Trends

#### Medical Devices

- *Medical devices* expenditure had an ascendant 20-year evolutionary trend (KPSS 0.085, CI 99%).
- Its share of the total SHI expenditure was below 1% and reflects an increase in the allocation of funds, with new devices available in the market where a portion of the costs are covered as co-payments by the patient.

#### Sanitary Materials From NHP

- *Sanitary materials from NHP* expenditure had an ascendant 20-year evolutionary trend (KPSS 0.138, CI 95%).
- Its share of the total SHI expenditure was around 1% with a maximum of 1.6% in 2016 (significant breakpoint, *p* < 0.05).

#### Dialysis

- *Dialysis* expenditure has an ascendant 20-year evolutionary trend (KPSS 0.190, CI 99%).
- However, after a continuous growth in budget share, from just over 1% in 2002 to a maximum of over 4% in 2015 (significant breakpoint, *p* < 0.01), the expenditure for *Dialysis* decreased to 3.1% in 2019.

#### Home Care Services

- *Home care services* expenditure has an ascendant 20-year evolutionary trend (KPSS 0.124, CI 95%).
- It represented a very small share in the categories of expenditure with a maximum of 0.3% of the total SHI expenditure in 2014 and 2015 (highly significant breakpoint, *p* < 0.001).

#### Medical Services Received in EU

**Figure 2:**
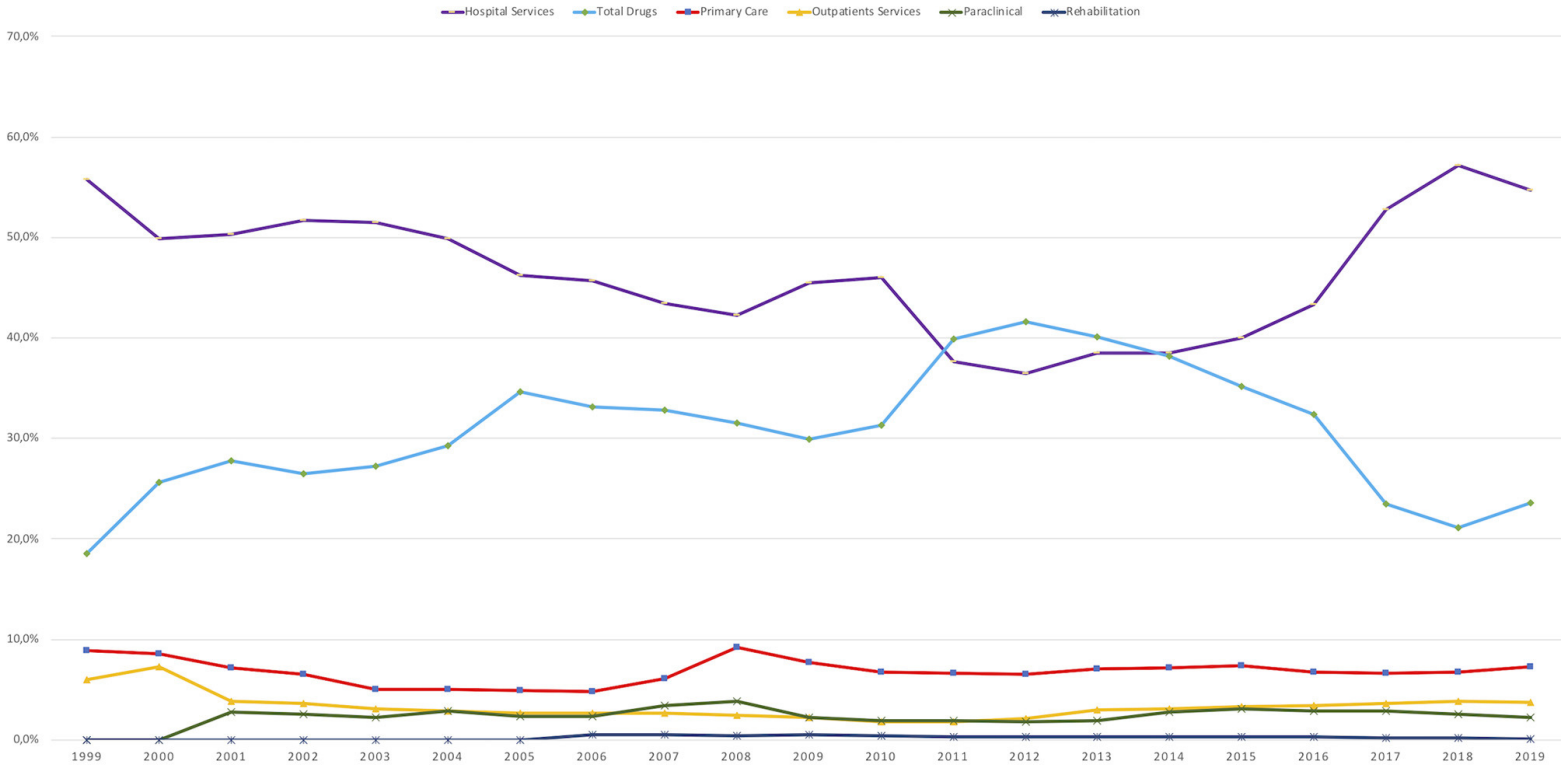
Categories of services with stationary trends, 1999–2019.

**Figure 3:**
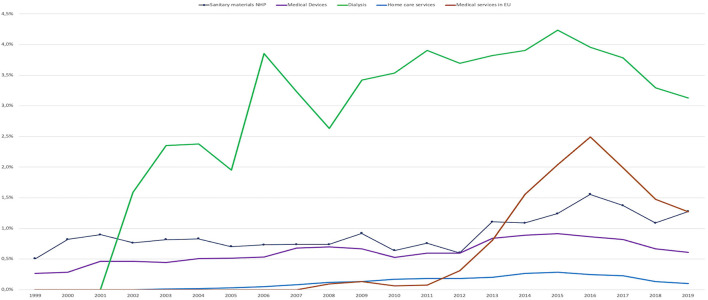
Categories of services with ascendant trends, 1999–2019.

- *Medical Services received in EU* expenditure has an ascendant 20-year evolutionary trend (KPSS 0.130, CI 95%).
- This category reached a maximum of 2.5% of the NHIH budget in 2016 and decreased thereafter (significant breakpoint, *p* < 0.05).
- The absolute amount, during the 2016 peak year, for *Medical Services received in EU* represents ~125 million Euro. This amount represented medical services imported to Romania from EU providers. During the period between 2008 and 2019 the total absolute amount paid from Romania to cover the *Medical Services received in EU* was over 650 million Euro and during the same period over 10,000 doctors left Romania. This movement of funds outside Romania, correlated with the migration of health personnel, and reflects the inability of the system to manage its resources correctly and efficiently causing a detriment in the health of the population and its own sustainability (Figures 2, 3).

### Categories of Services With Descendent Trends

#### Multifunctional Medical Centers

- An analysis on the *Multifunctional medical centers* expenditure revealed that the 20-year evolutionary trend was descendent (KPSS 0.076, Cl 99%).
- Their share in the total SHI expenditure was below 1% and reflected the low allocation of funds toward these centers, which have a role in allowing the continuity of care in out-patient settings.

#### Dentistry

- An analysis on the *Dentistry* expenditure revealed that the 20-year evolutionary trend was descendent (KPSS 0.199, Cl 99%).
- In 1999, the *Dentistry* category was 2.4% of the budget, but decreased to 0.3% in 2019 (significant breakpoint in 2013, *p* < 0.01). This could be due to pressure by other medical service categories for funds and social acceptance that dental services can be paid for from the patient's pocket. This left the population with low income without cover for dental care, which is reflected as an increase in general oral health issues of the population (13).

#### Emergency Services

- An analysis on the *Emergency services* expenditure revealed that the 20-year evolutionary trend was significantly descendant (KPSS 0.129, CI 95%).
- *Emergency services* expenditure dropped dramatically from 3.5% in 2012 to 0.2% share in 2014 due to a change in financing source, from the SHI budget to the Ministry of Health budget (significant breakpoint in 2013, *p* < 0.01).

#### Administrative Expenditure

**Figure 4:**
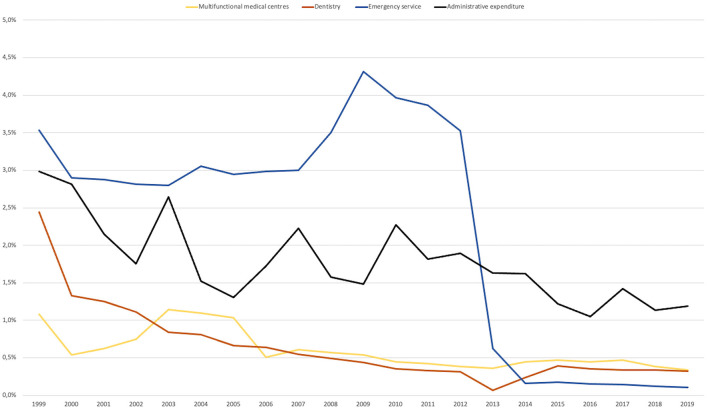
Categories of services with descendent trends, 1999–2019.

- This category had a significantly descendent evolutionary trend (KPSS 0.097, Cl 99%), from a height of ~3% to a little over 1% in recent years, even though the maximum percentage was set at 5% by the initial Social Health Insurance Law #145/1997.
- The *Administrative* expenditure category is related to the monitoring of the efficiency and quality control of the health system. This low level of administrative expenditure shows the shortcomings that arise especially in the functioning of the SHI informatics system and reflects the SHI and its personnel efforts to keep the SHI working with such few resources (Figure 4).

## Discussion

Our analysis covers only the curative services funded by the SHI scheme. However, the absolute public expenditure for health also includes other specific Ministry of Health expenditures such as that for national preventive programs, public health actions, and subordinate institutions with attributions related to public health (which represent about 12–18% of SHI budget, depending on the applicable year).

Additionally, the health expenditures of the local authorities were introduced from 1993 with a contribution of 16–18% of total public expenditures, but they dropped to <1% after the introduction of the Social health Insurance in 1999 (2). These local authorities' health expenditures become a more important share in health financing through the Framework Law on decentralization # 195/2006, which allowed the Local Authorities (LA) to devote a “Health” budget. The LA health budget was directed toward: “*medical services in health units with beds in general hospitals and medico-social units*,” “*public health services*,” “*other expenses for health*,” and “*other health institutions and health activities*” (14).

Starting from 2006, health expenses of LA grew by over 9 times, from 38 million Euro in 2006 to a maximum of 344 million Euro in 2015, representing ~8% compared to SHI budget. Subsequently, these expenses fell to 260 million Euro in 2016 representing 5% of SHI budget.

The views and commentaries on health expenditures in literature are commonly made on budget categories as is described in section “Methods” or using the System of Health Accounts (SHA).

The SHA represents a statistic tool to describe the financial flows related with provision of health care services. The first version of the SHA methodology was developed in 2000 (SHA1.0) by experts from the Organization for Economic Cooperation and Development (OECD). It is important to develop a harmonized SHA across international communities because of the apparent complexity and differences in the national health care systems, development of rapid medical techniques, growing requirements of medical service users, to initiate better public policies (15).

The last form of SHA available for Romania published by the National Institute of Statistics in December 2020 is related to 2018 data (15). SHA have a different classification for types of health providers, and a classification for types of services and sources of funding (public and private sources). We could not create an overlap between the analysis of the data used in this paper (expenditure data published by the NHIH about the SHI budget) and the data in the SHA.

The limitation of this study is that unlike the SHA method, the method of analysis used does not allow for comparisons with other countries (especially in the EU).

We had to adjust the expenditure categories for analysis in our study because the simple analysis of the expenditures categories published by SHI provided a distorted picture, since categories that belong to the same group were treated separately (e.g., *Transfers to public units* which are in fact hospital personnel salaries and should be viewed under *Hospital service*s category).

The 20-year evolutionary trends (1999–2019) for *Hospital services, Total drugs, Primary care, Outpatient services*, and *Paraclinical (laboratory) services* expenditure are stationary even though there were significant breakpoints during the study period, reflecting the auto-adjustment of the system.

Changes in the trends are seen only in the below 5% budget share categories, which reflects either market opportunities or a lack of priority placed on these care providers over the last two decades.

The fact that the major categories of expenditures were unchanged after 20 years, raises a question on the success of the structural changes in the system that imply expanding primary care and outpatient sector and diminishing tertiary care as foreseen in the Romanian National Strategy for Health 2014–2020 (12).

As other studies show, Romanian health budget had one of the biggest increases in the period of 1995–2014 (16) in the Central, East Europe Region, and the health spending is projected to continue increasing well into the future (17). The model of health care financing mechanisms appears not to be a key driver for raising healthcare costs in the Balkan region (18). Therefore, the focus goes on the quantity, equity, and efficiency of the health financing influenced by reforms in order to achieve improvement of the health outcomes.

Because the research covers a long period, the analysis considered to the trend of shares for each type of expenditures and not the absolute values of the expenditures, in order to avoid any discounting aspect.

Meantime, just to clear up once more for the reader, this is not an exhaustive analysis of the health care financing trends in Romania, but it is limited only to Social Health Insurance expenditures that covers only 65% of the total health care expenditure in 2019.

We consider that our research would be a good starting point for other studies in this area, to evaluate how different health or general financing policies (like the unique value for income tax) influenced the trends presented here, and compare trends in financing and the ones in volume of services provided in the same period.

## Conclusions

Financing of the Romanian health system has increased eight times over the last 20 years. In the same time, the percent of salaries paid as SHI contributions dropped from 14 to 10%. The local authorities are increasingly involved in financing the health system and supplementing the total budget with local funds.

The adjusted statistical analysis shows there were no significant structural changes in budget shares for the main categories. This reveals that there were no significant changes in the services structures: hospitals vs. primary care and outpatient services and this raises a question on the success of the planned strategic reforms.

The advantage of the method used in this paper is that it is in line with the historical planning and reporting of SHI financial data. We have adjusted each type of the allocated expenditure to the corresponding category and year. Thus, the trends reflect the real magnitude of each service as a component of the system. The statistical tools used increase the evidence of the findings.

## Data Availability Statement

The original contributions presented in the study are included in the article/supplementary material, further inquiries can be directed to the corresponding author.

## Author Contributions

C-PR: conceptualization, methodology, original draft preparation, reviewing and editing, and validation. BP: data curation, investigation, conceptualization, methodology, and writing—reviewing and editing. DP: statistic analysis. RC: conceptualization, methodology, and supervision. All authors contributed to the article and approved the submitted version.

## Conflict of Interest

The authors declare that the research was conducted in the absence of any commercial or financial relationships that could be construed as a potential conflict of interest.

## Publisher's Note

All claims expressed in this article are solely those of the authors and do not necessarily represent those of their affiliated organizations, or those of the publisher, the editors and the reviewers. Any product that may be evaluated in this article, or claim that may be made by its manufacturer, is not guaranteed or endorsed by the publisher.

## Footnotes

^1^https://ec.europa.eu/eurostat/web/health/data/database (accessed November, 2021).
