# Erratum: Evolution of Public Health Expenditure Financed by the Romanian Social Health Insurance Scheme From 1999 to 2019

**DOI:** 10.3389/fpubh.2022.857426

**Published:** 2022-02-15

**Authors:** Frontiers Production Office

**Affiliations:** Frontiers Media SA, Lausanne, Switzerland

**Keywords:** financing, trends, health system, health budget, hospital budget, drug expenditure

Due to a production error, the captions for Figures 2–4 were mistakenly written as:

Figure 2. Categories of services with ascendant trends, 19992019.

Figure 3. Categories of services with ascendant trends, 19992019.

Figure 4. Categories of services with descendent trends, 19992019.

The corrected captions for Figures 2–4 appear below.

Figure 2. Categories of services with stationary trends, 1999–2019.

Figure 3. Categories of services with ascendant trends, 1999–2019.

Figure 4. Categories of services with descendent trends, 1999–2019.

The publisher apologizes for this mistake. The original version of this article has been updated.

